# Knowledge and Perceptions of Human Papillomavirus Infection and Vaccination Among Adolescents in French Rural Family Homes: A Mixed-Methods Study

**DOI:** 10.3390/vaccines14090752

**Published:** 2026-08-29

**Authors:** Nolwenn Le Stang, Nicolas Palierne, Stéphanie Mignot, Gautier Defossez, Pierre Ingrand, Isabelle Ingrand

**Affiliations:** 1 Poitou-Charentes General Cancer Registry, CHU de Poitiers, Université de Poitiers, CIC-INSERM, axe SCALE-EPI, F-86000 Poitiers, Franceisabelle.ingrand@univ-poitiers.fr (I.I.); 2 Groupe de Recherches Sociologiques sur les sociétés Contemporaines, Université de Poitiers, F-86000 Poitiers, France; nicolas.palierne@univ-poitiers.fr; 3 Department of General Practice, University of Poitiers, Clinical Research Center CIC1402, INSERM, F-86000 Poitiers, France; stephanie.mignot@univ-poitiers.fr; 4 Centre for Primary Care and Public Health (Unisanté), University of Lausanne, 1010 Lausanne, Switzerland; gautier.defossez@unisante.ch

**Keywords:** human papillomavirus, HPV vaccination, adolescents, vaccine hesitancy, health education, vaccination uptake, rural populations

## Abstract

Background/Objectives: Human papillomavirus (HPV) causes several cancers in both females and males. In France, HPV vaccination coverage among adolescents remains suboptimal, particularly among boys, and knowledge about HPV infection and vaccination is limited. This study assessed HPV-related knowledge, attitudes, and vaccination uptake among adolescents attending Rural Family Homes (RFHs). Methods: A sequential mixed-methods study was conducted between September 2023 and June 2024 among 895 adolescents aged 15–19 years attending 19 RFHs in France. Participants completed an anonymous self-administered questionnaire assessing sociodemographic characteristics, general vaccination knowledge, HPV-related knowledge, vaccination status, and determinants of vaccine uptake. Focus groups involving 59 adolescents explored perceptions of, barriers to, and information needs regarding HPV vaccination. Quantitative data were analyzed using chi-square tests and multivariable logistic regression; qualitative data were analyzed thematically. Results: Overall, 44% of participants had received at least one HPV vaccine dose and 31% received at least two doses. Coverage was significantly higher among girls than boys (55% vs. 28%, *p* < 0.001). Only 35% demonstrated adequate HPV-related knowledge. Parents and general practitioners were the main information sources. Vaccination uptake was associated with favorable subjective norms, female sex, and the belief that vaccination reduces cancer risk. Qualitative findings revealed persistent misconceptions about HPV-related risks, particularly among boys, and a substantial need for reliable information. Conclusions: Adolescents attending RFHs showed limited HPV-related knowledge and suboptimal vaccination coverage, particularly boys. Parental support and healthcare professional recommendations were associated with vaccine uptake. Targeted educational interventions and catch-up vaccination strategies among this population are needed to improve coverage and reduce the burden of HPV-related diseases.

## 1. Introduction

Human papillomavirus (HPV) infection is one of the most common sexually transmitted infections worldwide. In both females and males, HPV can cause a wide range of conditions, from genital warts to several types of cancer. These complications do not occur immediately, as HPV-related lesions and cancers may take years to develop [1].

Although HPV infection is a necessary cause of nearly all cervical cancers (99.7%), it is also responsible for most anal cancers (90%), a substantial proportion of oropharyngeal cancers (35–63%), and penile cancers (60%). While cervical cancer remains the most common HPV-related cancer, the overall number of other HPV-associated cancers now exceeds that of cervical cancer, and several of these cancers affect males. In particular, the incidence of oropharyngeal cancer is higher among men and continues to increase, while the incidence of anal cancer is rising in both sexes [2,3].

Countries that have implemented HPV vaccination programs have demonstrated their effectiveness at the population level in reducing HPV infections and precancerous lesions [4]. Within 5–8 years of vaccine introduction, HPV prevalence decreased by 83% among girls aged 13–19 years, while the prevalence of precancerous cervical lesions declined by 51% among girls aged 15–19 years [5,6,7]. Increasing vaccination coverage is a global challenge to reduce HPV-related morbidity and mortality: currently estimated at 31% for the first dose of the HPV vaccine, it remains well below the 90% target set by the WHO for 2030 [8].

This burden is also substantial at the national level: in France, approximately 6400 new cancer cases attributable to HPV are diagnosed each year, with roughly three-quarters occurring in women and one-quarter in men. Cervical cancer accounts for the largest share, with just over 3100 new cases and 1100 deaths recorded annually, while anal and oropharyngeal cancers each account for approximately 1500 HPV-attributable cases per year [9], according to Santé publique France.

In France, a country without a national immunization program, mandatory infant vaccinations expanded substantially in 2018, from three mandatory vaccines (diphtheria–tetanus–polio) to 11, adding pertussis, *Haemophilus influenzae* type b, hepatitis B, pneumococcus, meningococcus C, measles, mumps, and rubella [10]. The HPV vaccine—the nonavalent formulation since 2018—has remained recommended, not mandatory, for children aged 11–14. Vaccines’ costs are covered at 65% by the government and 35% by supplemental health insurance (held by 97% of the population). For the HPV vaccine, 100% coverage has been available since 2023 when administered by a school nurse, but only in Year 8 (UK) or 7th grade (US) [11].

HPV vaccination policy has evolved gradually: recommended for 14-year-old girls in 2007, extended in 2013 to girls aged 11–14 years with catch-up vaccination up to age 19, and extended in 2016 to men who have sex with men [2]. In 2019, the French National Authority for Health (HAS) recommended extending vaccination to boys as part of a gender-neutral strategy [12], formally incorporated into the national immunization schedule in 2021; catch-up eligibility was further extended to age 26 in 2025. This progression reflects a shift from a herd-immunity strategy centered on girls toward universal vaccination grounded in individual benefit, reduced transmission, and equity. A complete HPV vaccination schedule consists of two doses for individuals aged 11–14 years and three doses for those aged 15 years or older.

Population-level data on HPV prevalence and precancerous lesion trends comparable to those observed in high-coverage countries are lacking in France, reflecting insufficient vaccination coverage to generate a measurable herd effect. However, individual-level effectiveness has been demonstrated: among sexually active young French women, HPV vaccination showed 95.9% effectiveness against vaccine-type genotypes (95% CI: 90.2–98.3) [13]. Nevertheless, vaccination coverage remains lower than that of other recommended vaccines and falls well below national targets, likely because it relies on vaccinations that have traditionally been administered in doctors’ offices [14]. In 2021, coverage was estimated at 46% for at least one dose among 15-year-old girls and 41% of 16-year-old girls had completed the three-dose schedule, while only 6% of boys of the same age had received at least one dose [15]. Adolescents aged 15–19 years therefore represent an important target population, particularly boys, many of whom remain unvaccinated [16]. As adolescents begin to explore romantic relationships and sexual activity [17], they may be particularly receptive to information regarding the benefits of HPV vaccination.

Previous studies have highlighted the central role of parents in decisions regarding adolescent vaccination [18]. However, socioeconomic disparities influence attitudes toward HPV vaccination, with less favorable perceptions observed among families with lower educational attainment and those living in disadvantaged areas [19,20,21].

Rural Family Homes (RFHs) [22], a network of approximately 430 educational institutions under the Ministry of Agriculture, may provide a relevant setting in which to address these inequalities. The enrollment of over 65,000 students (roughly 1% of French adolescents) involves little-to-no academic selection. Most operate as boarding schools, with about 80% of students residing on-site, and follow a work–study model alternating classroom instruction with practical training in local businesses. These institutions serve socially and educationally diverse populations, including adolescents experiencing academic difficulties, and are predominantly located in rural areas.

Knowledge and acceptance of HPV vaccination are key determinants of vaccine uptake [23], but they vary according to sociodemographic characteristics [24]. To date, no data are available on adolescents attending RFHs. This study therefore aimed to assess knowledge of HPV infections and vaccination, attitudes toward HPV vaccination, vaccination status, and associated factors among these adolescents, prior to the implementation of an educational intervention supported by the distribution of a comic book (CB).

## 2. Methods

### 2.1. Study Design and Population

An explanatory sequential mixed-methods study [25], combining a cross-sectional questionnaire survey and focus groups, was conducted between September 2023 and June 2024 among adolescents aged 15–19 years attending participating RFHs. The qualitative component was not designed to establish causal or temporal relationships, but rather to provide explanatory context for the quantitative associations observed, helping to interpret how and in what circumstances these variables were related, based on participants’ own accounts. The study protocol has been described in detail in a separate publication dedicated to this topic [26]. Briefly, in this article, we analyze the responses provided by adolescents through a dedicated questionnaire administered prior to any educational intervention related to our topic (questionnaire T0 in the protocol). We compare these results with the explanations given during the focus groups by the adolescents from the group unaware of any educational intervention (Group I in the protocol).

This questionnaire was developed for this study between September and December 2022, through a multidisciplinary collaboration—Maternal and Infant Protection (MIP) doctors and midwives, and RFH managers and health referents. It was based on validated models from this field: the Health Belief Model (HBM), which comprises seven cognitive constructs [27,28]—perceived susceptibility, perceived severity, perceived benefits, perceived barriers, cues to action, incentives to action, and self-efficacy —and the Theory of Planned Behavior (TPB) [29], which assumes that individuals act rationally according to their attitudes, subjective norms, and perceived behavioral control. It was tested on a panel of ten adolescents not enrolled in RFHs.

The study was presented to the parents of adolescents at the RFHs during a back-to-school meeting in September 2023 to explain its methodology and objectives and to obtain their consent. For adolescents of separated parents, both parents had to sign a consent form for their child to participate or, in the absence of parents, the legal guardian. It was then presented again to the adolescents by the RFHs’ health coordinators prior to the start of the study.

Participants were enrolled in Seconde (Year 11 in UK, Grade 10 in USA), Première (Year 12 or Grade 11) and CAP programs (Certificate of Professional Aptitude in France, a French vocational qualification) in 19 participating RFHs, representing a sample of 1385 students. All adolescents were eligible for inclusion regardless of their HPV vaccination status; those who had already received HPV vaccination were not excluded from the study.

### 2.2. Data Collection

#### 2.2.1. Quantitative Data Collection

Adolescents completed an anonymous self-administered questionnaire during class time between September and December 2023, according to each institution’s organizational procedures. This questionnaire was designed to assess HPV-related knowledge, vaccination status, and determinants of vaccine uptake before the implementation of any educational intervention. Vaccination status was determined by the following questions: “Have you been vaccinated against human papillomavirus?” and, if so, “How many doses have you received?”. It included the following sections:

- Sociodemographic characteristics;
- General knowledge of vaccination;
- Sources of health-related information;
- Knowledge of HPV infection and vaccination:
  Q1. “Do you think HPV is contagious?”—Yes, No, Don’t know.
  Q2. “In which sex can HPV cause health problems?”—Among women and men, Only in women, Don’t know.
  Q3. “How is HPV transmitted?”—Multiple-choice question: Ordinary physical contact (shaking hands), Sexual intercourse, While using the restroom, Through the air, Don’t know.
  Q4. “Can HPV cause cervical cancer?”—Yes, No, Don’t know.
  Q5.” Can HPV cause cancers other than cervical cancer?”—Yes, No, Don’t know.
  Q6. “Can HPV cause anal or genital warts?”—Yes, No, Don’t know.
  Q7. “The HPV vaccine is intended only for girls?”—True, False, Don’t know.
  Q8. “The HPV vaccine is safe”—True, False, Don’t know.
  Q9. “I’m too young to get the HPV vaccine”—True, False, Don’t know.
  Q10. “The HPV vaccine is intended only for sexually active individuals”—True, False, Don’t know.
- Self-reported health and vaccination history;
- Willingness to receive HPV vaccination;
- Determinants of HPV vaccination intention and uptake based on the Theory of Planned Behavior, including attitudes (e.g., perceiving HPV vaccination as beneficial) and subjective norms (e.g., perceived expectations of parents, physicians, and peers regarding vaccination).

#### 2.2.2. Qualitative Data Collection

Focus groups were conducted in two RFHs in June 2024, after completion of the questionnaire survey: the four focus groups included 59 adolescents (47 girls and 12 boys) enrolled in SAPAT (*n* = 21) or CAP (*n* = 38) programs.

Participation in the focus groups was offered at random to students who had not previously taken part in any intervention, with random selection nonetheless ensuring a representative sample in terms of gender and vocational track. Each session lasted approximately 60 min and was moderated by a sociologist assisted by one or two medical residents. Semi-structured interview guides were developed based on findings from the quantitative phase and the relevant literature.

Discussions explored participants’ knowledge and perceptions of vaccination, sources of information, preferred communication channels, vaccination experiences, and expectations and information needs regarding HPV vaccination.

The focus groups included both girls and boys who had not previously received any specific educational information about HPV infection or vaccination.

### 2.3. Data Analysis

#### 2.3.1. Quantitative Analysis

Adolescents who had received at least one dose of HPV vaccine were considered engaged in the vaccination process and were compared with unvaccinated adolescents. Complete vaccination coverage was defined as receipt of at least two doses, to account for the age-dependent variation in vaccination schedules at the time of the first dose.

A composite knowledge score was calculated by summing correct responses to the HPV-related knowledge questions described before. A specific HPV knowledge score was derived from a 10-item scale. Knowledge levels were categorized as follows:

(a) Adequate knowledge: ≥7 correct answers out of 10;
(b) Moderate knowledge: 5–6 correct answers;
(c) Inadequate knowledge: <5 correct answers.

Attitudes toward HPV vaccination were considered “favorable” when adolescents agreed with three statements regarding vaccine effectiveness against HPV infection, HPV-related cancers, and anal or genital warts. Subjective norms were assessed based on perceived expectations from parents, healthcare professionals, and friends regarding HPV vaccination. Among unvaccinated adolescents, intention to receive HPV vaccination was considered positive when participants reported being “*somewhat likely*” or more likely to accept vaccination.

The main outcome variables were HPV-related knowledge level and HPV vaccination status. Independent variables included sociodemographic characteristics (age, educational level, and educational orientation), sources of information, HPV-related knowledge, attitudes toward HPV vaccination, and subjective norms. Educational level was defined as CAP, Year 11 or Year 12, and educational orientation as Agronomy (comprising landscaper, farm machinery operator, stable hand), Marketing (comprising marketing/sales and management), or Services (comprising personal and local services (SAPAT) and catering). The sources of information were defined as parents/family, general practitioner, specialist doctor, other health professional, school (doctor, nurse, education program), peers, television, radio, internet, newspaper/magazine, other sources of information, or no specific source.

Continuous variables are presented as means and standard deviations, whereas categorical variables are presented as frequencies and percentages. Group comparisons were performed using Wilcoxon and Kruskal–Wallis tests for continuous variables and chi-square tests for categorical variables. Statistical significance was defined as *p* < 0.05.

Binary logistic regression analyses were performed to estimate crude odds ratios (ORs) and 95% confidence intervals (CIs) for associations between independent variables and outcomes of interest. Variables associated with the outcome at *p* ≤ 0.20 in univariate analyses were entered into multivariable logistic regression models after assessment of collinearity. Adjusted odds ratios (aORs) with 95% CIs were calculated. Age was systematically included as an adjustment variable. Variable selection was performed using a backward stepwise procedure with a significance threshold of 0.05. Potential interactions between variables were explored in the final models.

All statistical analyses were performed using SAS software version 9.3.

#### 2.3.2. Qualitative Analysis

Focus groups were audio-recorded with participants’ consent and transcribed verbatim in anonymized form by the same sociologist. Data were analyzed using thematic analysis [30]. Transcripts were read repeatedly to ensure familiarity with the data, and initial codes were generated inductively from the transcripts. The researchers read the transcripts independently. Codes and emerging themes were discussed during research team meetings to refine the coding framework and ensure consistency in data interpretation. The study was conducted and reported using the Consolidated Criteria for Reporting Qualitative Research (COREQ) [31]. No software was used to create these transcripts.

### 2.4. Ethical Aspects

The study protocol was approved by a French ethics committee (Comité de Protection des Personnes, CPP). Adolescents, parents, and teachers received an information letter describing the study objectives and procedures, together with a consent form. Prior to participation, oral informed consent was obtained from adolescents, and written parental non-opposition was obtained.

## 3. Results

A total of 895 adolescents aged 15–19 were included from 19 RFHs and 67 classes. The overall participation rate was 64%, ranging from 17% to 97% across institutions. Among participants who provided their sex (*n* = 877), 514 were girls (59%) and 363 were boys (41%), with a mean age of 16 ± −1 years. Most adolescents lived in rural (67%) or semi-rural (21%) areas. Participants were enrolled in CAP programs (25%), Year 11 (34%) or Year 12 (41%), and nearly half (48%) were studying personal and community services (SAPAT) or catering. Overall, 71% reported being up to date with their vaccinations (Table 1).

### 3.1. General Knowledge About Vaccination

Adolescents generally held positive views toward vaccination. Participants believed that major advances in vaccinology are still to come and that new vaccines will continue to be developed (mean score: 74/100). Vaccination was also widely perceived as a major medical achievement (75/100).

Most participants knew that vaccines are available for tetanus (90%), influenza (83%), and measles (70%), while 75% correctly identified that no vaccine exists for obesity.

However, substantial knowledge gaps were identified. Nearly three-quarters of adolescents did not know whether a vaccine against poliomyelitis existed (70%), while almost half were unaware of the existence of vaccines against hepatitis B (48%) and meningitis (51%). In contrast, 52% incorrectly believed that a vaccine against HIV/AIDS exists.

Overall, 58% of adolescents knew that a vaccine against cervical cancer was available. Awareness was significantly higher among girls than among boys (69% vs. 44%, *p* < 0.001) and among adolescents enrolled in SAPAT programs compared with those enrolled in other educational programs (65% vs. 53%, *p* = 0.01) (Table 2).

### 3.2. Sources of Vaccination-Related Information

#### 3.2.1. Vaccination in General

The main sources of information were parents or family members (91%) and general practitioners (GPs) (85%), followed by school-based sources (doctors, nurses, and educational programs) (44%), the media (internet: 38%, television: 36%) and peers (19%) (Table 3). Very few adolescents (4%) reported not having received information from either their parents or their GPs.

#### 3.2.2. HPV Vaccination

During the focus groups, adolescents reported that information about the HPV vaccine, as well as vaccination more generally, primarily came from family discussions (most often with mothers or female relatives, particularly those in healthcare-related professions), consultations with their GP, or conversations with peers.

*‘She [my mum] told me it was a vaccine against cervical cancer and that I had to get vaccinated straight away. Then she spoke to my brother about it, because men can transmit it to women. So my brother went to get vaccinated as well.’*

(vaccinated girl, 16)

While some parents expressed favorable views toward HPV vaccination, others opposed vaccination for their children, even when the adolescents themselves were in favor of being vaccinated.

*‘I haven’t had it yet, but I think it would be a good idea. I’m in favor of it. However, I also listen to my parents’ opinion. They’re not very keen on vaccines, including some non-mandatory ones, which they themselves haven’t had. So, I follow their advice because I trust their judgement.’*

(unvaccinated boy, 17)

In general, discussions with GPs took place in the presence of parents, who often provided additional explanations. Adolescents primarily understood HPV vaccination as an age-based recommendation intended to protect against future health risks. Several participants reported being prescribed the vaccine with little or no explanation from their physicians.

Sociologist: *‘Did she [GP] give you any additional information when you asked her about this vaccination?’*

Adolescent: *‘No.’*

Sociologist: ‘*No? She just prescribed the vaccine?*’

Adolescent: *‘Yes.’*

(vaccinated girl, 16)

However, one participant reported that his GP had not encouraged vaccination, while another explained that his mother had strongly opposed vaccination during the consultation without providing any further explanation.

*‘He [GP] said to me: “You could have it if you really had several partners, but otherwise you don’t need to.”’*

(unvaccinated boy, 17)

*‘One day, the doctor mentioned it to me because she thought it was a good idea, and then my mum said no, she definitely didn’t want me to have it.’*

(unvaccinated boy, 16)

Discussions with GPs often followed recommendations from parents. During the focus groups, girls frequently reported discussing vaccination with their female friends. These discussions focused less on the benefits of vaccination than on the pain associated with the injection.

*‘They [friends] told me it hurt, and that it was for vaginal cancer or something like that.’*

(vaccinated girl, 15)

Almost all adolescents reported having a GP (96%), but significantly more girls than boys had discussed HPV vaccination with their GP (62% vs. 35%, *p* < 0.001). Few adolescents had discussed the topic with another healthcare professional (13%), although this was significantly more common among girls than among boys (17% vs. 7%, *p* < 0.001).

### 3.3. General Knowledge About HPV-Related Diseases and the HPV Vaccine

During the focus groups, it became apparent that, apart from a few adolescents with a family history of cervical cancer, knowledge about HPV-related risks was often limited, vague, or inaccurate. In cases where there was a family history of HPV infection without cervical cancer, discussions sometimes focused on HPV itself without necessarily leading to a recommendation for vaccination.

*‘I was vaccinated as well. It was during a consultation with the GP who told me, when I was 13, that it would be a good idea for me to get vaccinated. My mother, um, had cervical cancer. She told me I should do it.’*

(vaccinated girl, 17)

Sociologist: ‘*So they [grandmother, great-grandmother, two aunts and mother] were all infected and told you that it could cause cancer, and that the papilloma could be removed before it developed further. Is that right? But they didn’t necessarily advise you to get vaccinated?*’

Adolescent: *‘No.’*

(girl vaccinated on the advice of her doctor, 16)

Many boys, and some girls, appeared unaware that HPV vaccination is recommended for boys.

Adolescent: *‘My mum talked about it with my sister, but I don’t know whether I was vaccinated or not […] She talked to me about it because I was there, but I wasn’t really listening.’*

Resident: ‘*Did you feel concerned by it*?’

Adolescent: *‘Not really, no.’*

(unvaccinated boy, 17)

*‘Personally, when I had it done, I didn’t know it was also for boys. The doctor told us about it. I think that for girls it’s well-explained, but for boys, it’s much… much less well explained.’*

(vaccinated girl, 16)

Knowledge of HPV-related risks for boys appeared to be more limited. Fewer boys knew that HPV can cause cervical cancer (45% vs. 63%, *p* < 0.001), that the vaccine is not intended exclusively for girls (14% vs. 19%, *p* = 0.007) or that they were not too young to be vaccinated (67% vs. 81%, *p* < 0.001) (Table 4).

During the focus groups, adolescents of both sexes were divided between those who viewed boys primarily as transmitters of the virus to girls, who could subsequently develop cervical cancer, and those who believed that boys themselves were also at risk of HPV-related diseases, particularly cancers. However, the specific diseases and cancer types involved were often not clearly identified.

*‘Men don’t have a uterus, so they can’t get it. But they can pass it on. I don’t know how they catch it, but I know they can pass it on to women.’*

(vaccinated girl, 16)

*‘At first I thought it only affected girls, but then I realised that it can affect boys too, and that it was important to get vaccinated.’*

(boy, 17)

Some parents emphasized to their children that the vaccine is not intended for boys.

*‘And my father, I don’t know if, um, he’s against vaccines or what, it’s a lack of knowledge, he said that it was useless, that it wasn’t the right approach. […] He said that it didn’t concern men.’*

(boy who wished to be vaccinated, 18)

Only 22% of adolescents believed that the HPV vaccine was safe (Table 4), and only 35% demonstrated an adequate level of knowledge about HPV vaccination and HPV-related infections (Table 5). Having discussed HPV vaccination with a GP was the only factor positively associated with HPV-related knowledge (*p* < 0.001).

Most adolescents (82%) knew that sexual intercourse is the primary mode of HPV transmission. In two groups of Year 12 students, most participants believed that condoms provided effective protection against all sexually transmitted infections, including HPV.

### 3.4. Vaccination Coverage Against HPV Infections

Overall, 31% of adolescents had received at least two doses (40% of girls vs. 18% of boys; *p* < 0.001), while 44% had received at least one dose (55% of girls vs. 28% of boys; *p* < 0.001). During the focus groups some adolescents reported initiating HPV vaccination after the study had been presented, suggesting that participation in the study may have increased parental awareness of the benefits of vaccination.

Vaccination was significantly more frequent among girls than among boys (*p* < 0.001), among adolescents who had discussed HPV vaccination with their GP and demonstrated adequate knowledge of HPV infections and vaccination (*p* < 0.001), among those enrolled in services- or marketing-related educational programs (*p* = 0.01), and among Year 11 students (*p* = 0.003). Notably, the belief that HPV vaccination reduces the risk of anal or genital warts was not associated with vaccination status.

In the multivariate analysis, the variables “Consultation with a doctor regarding HPV vaccination” and “Adequate knowledge of HPV infections and vaccination” were excluded because of strong collinearity. No interaction between variables was identified. Factors independently associated with HPV unvaccinated status included favorable subjective norms (i.e., the belief that parents, healthcare professionals, and friends thought adolescents should receive the HPV vaccine) (aOR 0.20, 95% CI 0.14 to 0.27), female sex (aOR 0.36, 95% CI 0.26 to 0.50) and the belief that HPV vaccination reduced cancer risk (aOR 0.70, 95% CI 0.50 to 0.98) (Table 6). In other words, these factors help protect against the risk of not getting vaccinated.

During the focus groups, some adolescents acknowledged that they were unsure whether they had been vaccinated. *‘It was my godmother, […] She’s a nurse. Well, she told me about it and I think she did it for me.’*

(vaccinated girl, 15)

Indeed, one quarter of adolescents did not know whether they were up to date with their vaccinations, and 18% did not know whether they had received the HPV vaccine (Table 1). Furthermore, although 20% believed that they were up to date with their vaccinations, they had not received HPV vaccination.

### 3.5. Adolescents’ Experiences with HPV Vaccination

During the focus groups, adolescents were asked about their experiences with HPV vaccination.

#### 3.5.1. Reasons for Hesitation or Apprehension Among Vaccinated Adolescents

For some adolescents, fear of injections was overcome only because of parental encouragement or insistence.

*‘As soon as I saw the needle, I backed away, and my mum grabbed me, […] then they gave me the injection, yeah, the injection, and then, well, the product, I thought to myself [inaudible].’*

(vaccinated girl, 16)

#### 3.5.2. Reasons for Accepting Vaccination

Although HPV vaccination was often recommended by healthcare professionals or encouraged by parents, some adolescents reported choosing to be vaccinated on their own initiative in order to protect themselves against cancer.

*‘I was the one who made the decision and asked my mum about it […] and when I found out it was about related to cancer [my friends] and everything, I said yeah, all these diseases scare me, I’d rather, um… get vaccinated. I talked to her [my mother] about it and she said she completely agreed and that, at my age, she would have done the same.’*

(vaccinated girl, 15)

*‘For me, what scares me most is that my mother [a healthcare professional] is seeing more and more cases of cancer. It really frightens me.’*

(vaccinated girl, 15)

Among some vaccinated adolescents, HPV vaccination was perceived as a routine measure or simply as “*vaccine like any other”*, with little expectation of receiving additional information or explanation.

*‘Well, for me it happened because I went to have a blood test before my operation and then they gave me the vaccine.’*

(vaccinated girl, 16)

### 3.6. Attitudes of Unvaccinated Adolescents

Only 42% of unvaccinated adolescents reported that they would seek information about HPV vaccination and consider being vaccinated, while approximately half (49%) stated that they would do so if a physician recommended it (Table 7).

Compared with vaccinated adolescents, unvaccinated participants were significantly less likely to hold favorable attitudes toward HPV vaccination. They were less likely to agree that vaccination could protect against HPV infection, reduce the risk of HPV-related cancers, or prevent anal or genital warts (48% vs. 70%). Vaccine cost also appeared to contribute to reluctance toward vaccination. Only a minority reported that they would still choose vaccination if it were expensive (28%), while approximately half mentioned fear of injection-related pain (49%) or difficulties in arranging a medical appointment (44%) as potential barriers.

During the focus groups, these unvaccinated adolescents (more often boys than girls) appeared to be in either the precontemplation or contemplation stage with regard to HPV vaccination. According to the Transtheoretical Model of behavior change [32], precontemplation refers to having no intention of getting vaccinated within the next six months, often due to low awareness or risk perception, whereas contemplation reflects active consideration of vaccination without yet being ready to commit. They emphasized the importance of receiving advice and encouragement from trusted adults before making a decision. *‘No one really advised me to do it… Not even the family midwife, and, I don’t know, it doesn’t really help.’*

(girl, 16)

When adolescents were afraid of injections or did not feel personally concerned about HPV-related risks, the freedom granted by their parents to decide whether or not to be vaccinated could be sufficient for them to decline vaccination.

Adolescent: *‘My mum told me that if I want to do it, I can do it, and if I don’t want to, that’s fine too.’*

(girl, 16)

Sociologist: ‘*So your parents are giving you the choice, but you don’t want to do it.*’

Even when information was available, boys appeared to have greater difficulty perceiving themselves as personally concerned by HPV-related risks, particularly when they viewed HPV vaccination primarily as a vaccine for girls or when there was no family history of HPV-related disease.

*‘Yes. I had already heard about it, but I didn’t really feel concerned, um, or I didn’t have, um, anyone close to me, um… who had been infected’*

(boy, 18)

### 3.7. Information Needs

During the focus groups, adolescents repeatedly highlighted the limited involvement of healthcare professionals in discussions about HPV vaccination. Some felt that their questions had not been adequately answered and reported receiving insufficient information before vaccination decisions were made. Information provided by RFHs, families, and healthcare professionals was often perceived as incomplete or insufficient.

*‘Looking back, I think the course was perhaps a bit superficial. Because, OK, we learned about sexually transmitted diseases, but… we didn’t really cover everything. I don’t know how to explain it [smile].’*

(vaccinated girl, 16)

## 4. Discussion

The first finding of this study is that, despite a generally positive perception of vaccination, French adolescents attending RFHs remain poorly informed about the diseases against which they are, or could be, vaccinated. Too many were unaware that vaccines exist for poliomyelitis, hepatitis B, and meningitis, even though some of these have been mandatory for infants since 2018. In addition, a substantial proportion believed that a vaccine against HIV/AIDS exists, whereas no vaccine candidate is currently in phase III clinical trials. Consistent with previous studies [33], our findings highlight the need to improve HPV-related knowledge among adolescents attending RFHs.

Approximately half of the adolescents had received at least one dose of HPV vaccine, with a marked gender disparity (55% of girls vs. 28% of boys). These rates are close to those observed in the general population (55% of 15-year-old girls vs. 26% of boys) [12]. A nationwide school-based HPV vaccination campaign targeting adolescents aged 12–14 years was implemented during the 2023–2024 academic year, after completion of this study. Vaccination coverage among adolescents aged 15–19 years, who were not targeted by this campaign, therefore remains insufficient.

Given the substantial burden of HPV-related diseases and the accumulating evidence supporting vaccine effectiveness, the HAS recommended in 2025 that catch-up HPV vaccination be extended to unvaccinated young adults up to 26 years of age, regardless of sex or sexual orientation [34].

These adolescents, who are beginning their sexual lives, demonstrated limited knowledge about HPV infections and vaccination. As reported in previous studies, girls appeared to be better informed than boys [35,36]. This difference may reflect the fact that girls are more likely than boys to discuss sexuality, sexual health, contraception, and bodily changes with their mothers [37]. In addition, until 2021, HPV vaccination in France was recommended only for girls.

HPV vaccination uptake was associated with higher levels of HPV-related knowledge. Previous studies have similarly shown that limited knowledge is associated with a reduced understanding of the need for vaccination [38,39,40]. Our findings, particularly the observation that HPV-related diseases other than cervical cancer were largely unknown among both adolescents and parents, suggest that information campaigns should place greater emphasis on other HPV-related cancers, precancerous lesions, and the risks affecting boys.

As highlighted above, access to reliable, balanced, and understandable scientific information is likely to support informed decision-making. Adolescents who had discussed HPV vaccination with a physician were more likely to report better knowledge and greater acceptance of vaccination [38]. Consistent recommendations from healthcare professionals have repeatedly been shown to promote vaccine acceptance [41].

This study among adolescents attending RFHs highlights an association between physician involvement and HPV vaccination decision-making. Doubts expressed by some adolescents about vaccine effectiveness and potential side effects, as previously described [42], make discussions with healthcare professionals even more important [43,44]. Healthcare professionals should therefore place greater emphasis on discussions relating to adolescent health [45].

Several adolescents reported that their doctor had simply prescribed the vaccine without providing further explanation, while a few boys mentioned that their doctor had not actively encouraged them to receive HPV vaccination. Social media appeared to play a less prominent role than anticipated. Nevertheless, misinformation disseminated online may have contributed to vaccine hesitancy. Media-based information cannot replace face-to-face discussions with healthcare professionals [42].

Parental involvement is associated with vaccine uptake among adolescents, and it therefore seems necessary to involve them in programs promoting HPV vaccination, particularly since, in France, vaccination of minors requires parental consent [46]. Adolescents in this population also tend to trust their parents’ decisions regarding health matters. A review of the literature has highlighted the potential benefits of allowing adolescents to provide independent consent for HPV vaccination themselves, noting that countries with higher vaccination coverage often grant adolescents greater autonomy, particularly by allowing access to vaccination without parental authorization [47].

During the focus groups, several adolescents reported initiating HPV vaccination shortly after the study was presented to them, suggesting that participation in the study may have increased parental awareness of the benefits of vaccination and prompted vaccination decisions. This observation further supports the need for broader information campaigns targeting both adolescents and parents [48,49].

According to our findings, even adolescents with limited knowledge about HPV infections and vaccination appeared willing to learn more, suggesting that they perceived the topic as important. Their attitudes indicate that this population may be receptive to educational interventions about HPV vaccination. However, previous studies have consistently reported a gap between intention to vaccinate and actual vaccine uptake, with intention not always translating into behavior [50,51]. The lack of intervention by healthcare professionals was clearly regretted by adolescents in this study. At the same time, the information provided by RFHs was often perceived as insufficient, despite schools representing an important source of health information [52]. This study also showed that the importance given to sexual health prevention and awareness of legal obligations varied considerably between RFHs. The French Ministry of Education requires that at least three sex education sessions per year be delivered in secondary schools. RFHs, although funded by the Ministry of Agriculture, are subject to the same obligations, yet several institutions appeared unaware of this legal framework. RFHs should therefore be considered key settings for health promotion.

The strengths of this research lay in the combination of both quantitative and qualitative approaches and in the large number of female and male adolescents included. Focus-group interviews helped contextualize and enrich the findings obtained from the quantitative survey.

However, several limitations should be acknowledged. A further limitation concerns the heterogeneity of participation across RFHs and classes, which averaged 64% but ranged from 17% to 97% across schools. In some cases, entire classes did not participate, mainly because of scheduling conflicts with work placements or extended absence of the RFH health coordinator. As no comparison was possible between participating and nonparticipating students or classes, and as these groups cannot be assumed to be exchangeable, selection bias cannot be excluded. One of the main challenges concerned obtaining consent forms that were correctly completed and signed by both parents. Participation required active parental consent, which may have limited participation among adolescents whose families experienced difficulties completing administrative procedures. Parental consent procedures have previously been identified as a barrier to HPV vaccine uptake, particularly because of logistical difficulties associated with returning consent forms [53]. As in several previous studies, vaccination status was based on self-reported data [16], as the CPP did not authorize collection of anonymized photocopies of vaccination records, owing to the administrative burden this would have placed on RFHs. This raises concerns about validity: during focus groups, some adolescents acknowledged uncertainty about their own vaccination status, and indeed one quarter did not know whether they were up to date with their vaccinations, while 18% did not know whether they had received the HPV vaccine. Adolescents may not always be reliable informants regarding vaccines administered years earlier by a parental decision. Future studies should anticipate this constraint at the ethics application stage, for instance by requesting authorization to verify self-reported status against records for a subsample.

An additional limitation concerns the cross-sectional design of the quantitative component of this explanatory sequential mixed-methods study. Associations observed between HPV-related knowledge, attitudes, and vaccination uptake cannot be interpreted as causal, since exposure and outcome were measured simultaneously; reverse causality cannot be excluded, as vaccinated adolescents may have sought more information as a result of vaccination rather than knowledge having driven uptake. The qualitative focus groups similarly provide contextual and explanatory insight rather than evidence of causal mechanisms. Longitudinal designs would be needed to establish the temporal sequence between knowledge, attitudes, and vaccination decisions.

These findings suggest several practical directions. Healthcare professionals working with adolescents attending RFHs should move beyond simple prescription toward structured discussions of HPV vaccination, particularly with boys, and should be better informed of the legal obligation to deliver sex education in RFHs. Information campaigns targeting this population should involve parents alongside adolescents, given the central role of parental consent in the French context, while simplifying consent procedures may reduce a practical barrier to uptake. Future research should assess the impact of the 2023–2024 school-based campaign on adolescents aged 15–19 years, who were not directly targeted, and evaluate whether the recently recommended catch-up vaccination up to age 26 improves coverage. Longitudinal studies are also needed to clarify the gap between vaccination intention and actual uptake and to test whether school- and RFH-based interventions can close it.

## 5. Conclusions

This study highlights the limited knowledge about HPV infections and their prevention among adolescents attending RFHs, despite an overall positive perception of vaccination. These findings support the importance of knowledge, parental involvement and support from healthcare professionals in decision-making within this population. Results suggest that current information campaigns may not sufficiently emphasize the full range of HPV-related cancers or the health risks affecting boys as well as girls.

In this context, these findings support the need to strengthen and adapt awareness-raising initiatives aimed at adolescents attending RFHs and their parents, with a focus on reliable and accessible sources of information. The roles of physicians, schools, and educational institutions such as RFHs should be strengthened to ensure that adolescents receive comprehensive, evidence-based, and accessible information about HPV and its prevention. Investing in improved health education and appropriate prevention measures within RFHs is therefore an essential step towards protecting the long-term health of younger generations.

## Figures and Tables

**Table 1:** Characteristics of adolescents who responded to the questionnaire (*n* = 895).

	Overall
	Mean (sd)
Age, years (*n* = 851) *	16 (1)
	Number (%)
Gender (*n* = 877) *	
Girl	514 (59%)
Boy	363 (41%)
Habitat (*n* = 869)	
Urban	110 (13%)
Semi-rural	180 (21%)
Rural	579 (67%)
School level (*n* = 895)	
CAP	224 (25%)
Year 11	302 (34%)
Year 12	369 (41%)
Section (*n* = 842) **	
Agronomy	193 (23%)
Consulting/sales	250 (30%)
Services (personal and catering)	401 (47%)
The adolescent believes he is up to date with his vaccinations (*n* = 780)	
Yes	558 (72%)
No	33 (4%)
Don’t known	191 (24%)
The adolescent says he/she has been vaccinated against human papillomavirus (*n* = 883)	
Yes	394 (45%)
No	332 (37%)
Don’t known	157 (18%)

* A total of 44 adolescents did not fill in their age and 18 did not fill in their gender. ** Year 11 students from the General section are not counted.

**Table 2:** Adolescents’ knowledge of vaccination in general based on responses scored from 0, strongly disagree, to 100, strongly agree, and reported as mean (standard deviation).

Overall	By Gender		By School Level			By Main Career Focus		
	Girls	Boys	CAP	Year 11	Year 12	Services *	Agronomy	Marketing **
There are still important discoveries to be made in the field of vaccination, other vaccines to be discovered.								
*n* = 864	*n* = 491	*n* = 355	*n* = 216	*n* = 296	*n* = 352	*n* = 384	*n* = 186	*n* = 243
74 (22)	74 (22)	75.0 (23)	71(25)	77 (21)	74 (22)	74 (22)	74 (24)	75 (22)
	*p_a_* = 0.19		*p_b_* = 0.04			*p_b_* = 0.67		
Vaccination is truly a major discovery for health.								
*n* = 864	*n* = 492	*n* = 354	*n* = 213	*n* = 296	*n* = 355	*n* = 385	*n* = 185	*n* = 243
75 (22.7)	72 (22.5)	78 (22.6)	70 (25)	78 (21)	75 (22)	74 (23)	74 (24)	76 (22)
	*p_a_* < 0.001		*p_b_* = 0.003			*p_b_* = 0.43		
Vaccination is the best way to prevent disease because it is effective.								
*n* = 866	n = 494	n = 354	n = 216	n = 296	n = 354	*n* = 385	*n* = 187	*n* = 243
63 (24)	62 (24)	65 (24)	59 (26)	64 (24)	65 (23)	62 (25)	62 (24)	65 (24)
	*p_a_* = 0.01		*p_b_* = 0.007			*p_b_* = 0.35		
Vaccination is synonymous with innovation								
*n* = 838	*n* = 477	*n* = 343	*n* = 205	*n* = 287	*n* = 346	*n* = 372	*n* = 177	*n* = 238
58 (24)	56 (23)	62 (25)	53 (26)	62 (23)	59 (23)	56 (25)	60 (23)	61 (24)
	*p_a_* < 0.001		*p_b_* = 0.001			*p_b_* = 0.01		
There have been no major advances in the field of vaccination in the last 20 years.								
*n* = 850	*n* = 481	*n* = 351	*n* = 213	*n* = 287	*n* = 350	*n* = 377	*n* = 185	*n* = 237
42 (30)	43 (29)	41 (32)	47 (29)	43 (32)	39 (30)	43 (30)	45 (31)	40 (30)
	*p_a_* = 0.15		*p_b_* = 0.02			*p_b_* = 0.20		
Adolescents’ knowledge about cervical cancer by classes—response to the question Vaccines available for cervical cancer in France—number (%)								
	*n* = 508	*n* = 352	*n* = 217	*n* = 297	*n* = 363	*n* = 392	*n* = 189	*n* = 247
Yes	349 (69%)	155 (44%)	135 (62%)	156 (53%)	222 (61%)	255 (65%)	98 (52%)	134 (54%)
No	59 (12%)	93 (26%)	35 (16%)	63 (21%)	58 (16%)	57 (15%)	43 (23%)	46 (17%)
Don’t know	100 (20%)	104 (30%)	47 (22%)	78 (26%)	83 (23%)	80 (20%)	48 (25%)	67 (27%)
	*p_c_* < 0.001		*p_c_* = 0.14			*p_c_* = 0.01		

* Personal and Local Services (SAPAT) and catering; ** Marketing and Sales/Management; a, Wilcoxon two-sample test; b, Kruskal–Wallis test; c, Pearson chi^2^ test.

**Table 3:** Sources of information for adolescents regarding vaccination by adolescents’ sex.

	Overall (*n* = 895)	Girls (*n* = 514)	Boys * (*n* = 363)
Parents, family	811 (91%)	473 (92%)	323 (89%)
General Practitioner	761 (85%)	454 (88%)	293 (81%)
Specialist doctors	191 (21%)	110 (21%)	76 (21%)
Other healthcare professionals	164 (18%)	91 (18%)	71 (20%)
School (doctor, nurse, education program)	390 (44%)	214 (42%)	165 (45%)
Peers	166 (18%)	87 (17%)	74 (20%)
Television	322 (36%)	172 (33%)	142 (39%)
Radio	115 (13%)	37 (7%)	77 (21%)
Internet	336 (37%)	174 (34%)	154 (42%)
Newspapers/magazines	83 (9%)	35 (7%)	47 (13%)
Other sources of information	49 (5%)	28 (5%)	21 (6%)
No specific source	19 (2%)	7 (1%)	12 (3%)

* Eighteen adolescents did not report their gender.

**Table 4:** Knowledge about HPV-related diseases and vaccination; number (% by row).

		Yes	No	Don’t Know	Chi^2^ Test
Do you think HPV is contagious?	Overall	507 (57%)	122 (14%)	258 (29%)	
	Girls	266 (52%)			*p* < 0.001
	Boys	231 (64%)			
Can HPV cause cervical cancer?	Overall	498 (56%)	28 (3%)	360 (41%)	
	Girls	322 (63%)			*p* < 0.001
	Boys	163 (45%)			
Can HPV cause cancers other than cervical cancer?	Overall	237 (27%)	85 (10%)	560 (63%)	
	Girls	129 (25%)			*p* = 0.03
	Boys	102 (28%)			
Can HPV cause anal or genital warts?	Overall	165 (19%)	54 (6%)	666 (75%)	
	Girls	92 (18%)			*p* = 0.88
	Boys	69 (19%)			
Is the HPV vaccine only for girls?	Overall	85 (10%)	660 (74%)	141 (16%)	
	Girls			70 (14%)	*p* = 0.007
	Boys			69 (19%)	
Is the HPV vaccine safe?	Overall	194 (22%)	270 (31%)	419 (47%)	
	Girls	110 (22%)			*p* = 0.74
	Boys	81 (22%)			
I am too young to receive the HPV vaccine.	Overall	40 (4%)	671 (76%)	175 (20%)	
	Girls		414 (81%)		*p* < 0.001
	Boys		243 (67)		

**Table 5:** Determinants of overall knowledge scores on HPV-related diseases and vaccination, number (% by row).

	Adequate ≥ Seven Informed Questions	Moderate Five or Six Informed Questions	Inadequate ≤ Four Informed Questions	Univariate Analysis
	313 (35%)	315 (35%)	267 (30%)	
Gender				*p* = 0.17
Girls (*n* = 514)	187 (36%)	186 (36%)	141 (28%)	
Boys (*n* = 363)	119 (33%)	123 (34%)	121 (33%)	
School level				*p* = 0.44
CAP (*n* = 224)	75 (33%)	73 (33%)	76 (34%)	
Year 11 (*n* = 302)	100 (33%)	113 (37%)	89 (30%)	
Year 12 (*n* = 369)	138 (37%)	129 (35%)	102 (28%)	
Main career focus				*p* = 0.88
Services * (*n* = 401)	134 (33%)	140 (35%)	127 (32%)	
Agronomy (*n* = 193)	67 (35%)	70 (36%)	56 (29%)	
Marketing ** (*n* = 250)	82 (33%)	96 (38%)	72 (29%)	
Consultation with a doctor regarding HPV vaccination				*p* < 0.001
Yes (*n* = 413)	173 (42%)	163 (39%)	77 (19%)	
No (*n* = 421)	127 (30%)	136 (32%)	158 (38%)	

* Personal and Local Services (SAPAT) and catering; ** Marketing and Sales/Management.

**Table 6:** Determinants of HPV vaccination status: Has been vaccinated or is in the process of being vaccinated against papillomavirus infections (at least one injection); number (% by row).

		Analysis	
	Vaccinated or in the Process	Univariate	Multivariate
Sex		<0.001	<0.001
Girls (*n* = 514)	281 (55%)		Ref—
Boys (*n* = 363)	101 (28%)		0.36 [0.26–0.50]
School level		0.003	*NS ^a^*
CAP (*n* = 224)	84 (37%)		
Year 11 (*n* = 302)	156 (52%)		
Year 12(*n* = 369)	154 (42%)		
Main career focus		0.01	*NS ^a^*
Services * (*n* = 401)	194 (48%)		
Agronomy (*n* = 193)	68 (35%)		
Marketing ** (*n* = 250)	110 (44%)		
Consultation with a doctor regarding HPV vaccination		<0.001	Excluded
Yes (*n* = 413)	331 (80%)		
No (*n* = 421)	43 (10%)		
Level of knowledge of HPV infections and vaccination		<0.001	Excluded
Adequate (*n* = 313)	166 (53%)		
Moderate (*n* = 408)	189 (46%)		
Inadequate (*n* = 174)	39 (22%)		
ATTITUDE			
HPV vaccination protect me against genital infection by HPV		<0.001	*NS ^a^*
In agreement (*n* = 455)	236 (52%)		
Neutral or not in agreement (*n* = 440)	158 (36%)		
If I get vaccinated against HPV, I can reduce my risk of HPV-related cancers		<0.001	0.004
In agreement (*n* = 612)	298 (49%)		Ref—
Neutral or not in agreement (*n* = 283)	96 (34%)		0.70 [0.50–0.98]
If I get vaccinated against HPV, I will reduce my risk of developing anal or genital warts.		0.67	*NS ^a^*
In agreement (*n* = 284)	128 (45%)		
Neutral or not in agreement (*n* = 611)	266 (43%)		
SUBJECTIVE STANDARDS			
To what extent do [your parents, your doctor, your best friend] think you should receive the HPV vaccine?		<0.001	<0.001
In agreement (*n* = 394)	236 (70%)		Ref—
Neutral or not in agreement	158 (28%)		0.20 [0.14–0.27]
In general, I want to do what my parents, my doctor, and/or my best friend think I should do.		<0.001	*NS ^a^*
In agreement (*n* = 231)	132 (57%)		
Neutral or not in agreement (*n* = 664)	262 (39%)		

* Personal and Local Services (SAPAT) and catering; ** Marketing and Sales/Management; a. NS, non-significant.

**Table 7:** Attitude of adolescents who have not yet been vaccinated; number (% by row).

**I Am Confident in My Ability to Get Vaccinated Against HPV, Even If…**							
Self-efficacy	In complete disagreement	Disagree	Slightly disagree	Neutral	Slightly agree	Agree	In complete agreement
It is expensive (*n* = 475)	77 (16%)	38 (8%)	43 (9%)	185 (39%)	60 (13%)	53 (11%)	19 (4%)
The injection hurts a little (*n* = 475)	49 (10%)	22 (5%)	30 (6%)	141 (30%)	67 (14%)	89 (19%)	77 (16)
It means finding the time to go to the doctor three times (*n* = 478)	53 (11%)	28 (6%)	37 (8%)	149 (31%)	56 (12%)	83 (17%)	72 (15%)
**Plans for the coming year**							
Intentions	Highly unlikely	Unlikely	Slightly unlikely	Neutral	Slightly likely	Likely	Highly likely
How likely are you to seek further information about the HPV vaccine? (*n* = 470)	49 (10%)	39 (8%)	54 (12%)	131 (28%)	77 (16%)	74 (16%)	46 (10%)
What is the likelihood that you will actually get vaccinated against HPV? (*n* = 473)	71 (15%)	32 (7%)	40 (9%)	139 (29%)	63 (13%)	71 (15)	57 (12%)
If a doctor offered you the HPV vaccine in the next year, how likely would you be to get vaccinated? (*n* = 473)	62 (13%)	36 (7%)	32 (7%)	112 (24%)	66 (14%)	79 (17%)	86 (18%)

## Data Availability

The data presented in this study are available on request from the corresponding author.

## Abbreviations

CB	Comic book
HAS	French National Authority for Health
HBM	Health Belief Model
HPV	Human papillomavirus
MIP	Maternal and Infant Protection
RFH	Rural Family Home
TPB	Theory of Planned Behavior
